# Chest Compressions for Bradycardia during Neonatal Resuscitation—Do We Have Evidence?

**DOI:** 10.3390/children6110119

**Published:** 2019-10-29

**Authors:** Vikash Agrawal, Satyan Lakshminrusimha, Praveen Chandrasekharan

**Affiliations:** 1 Division of Neonatology, Department of Pediatrics, University at Buffalo, Buffalo, NY 14260, USA; vikashag@buffalo.edu; 2 Division of Neonatology, Department of Pediatrics, University of California Davis, Davis, CA 95616, USA; slakshmi@ucdavis.edu

**Keywords:** neonatal resuscitation, bradycardia, chest compressions

## Abstract

The International Liaison Committee on Resuscitation (ILCOR) recommends the initiation of chest compressions (CC) during neonatal resuscitation after 30 s of effective ventilation if the infant remains bradycardic (defined as a heart rate less than 60 bpm). The CC are performed during bradycardia to optimize organ perfusion, especially to the heart and brain. Among adults and children undergoing cardiopulmonary resuscitation (CPR), CC is indicated only for pulselessness or poor perfusion. Neonates have a healthy heart that attempts to preserve coronary and cerebral perfusion during bradycardia secondary to asphyxia. Ventilation of the lungs is the key step during neonatal resuscitation, improving gas exchange and enhancing cerebral and cardiac blood flow by changes in intrathoracic pressure. Compressing the chest 90 times per minute without synchrony with innate cardiac activity during neonatal bradycardia is not based on evidence and could potentially be harmful. Although there are no studies evaluating outcomes in neonates, a recent pediatric study in a hospital setting showed that when CC were initiated during pulseless bradycardia, a third of the patients went into complete arrest, with poor survival at discharge. Ventilation-only protocols such as helping babies breathe are effective in reducing mortality and stillbirths in low-resource settings. In a situation of complete cardiac arrest, CC reinitiates pulmonary flow and supports gas exchange. However, the benefit/harm of performing asynchronous CC during bradycardia as part of neonatal resuscitation remains unknown.

## 1. Introduction

Neonates that require extensive resuscitation involving chest compressions (CC) and epinephrine are at higher risk of neurological morbidity and mortality [1]. Unlike the adult or pediatric population, where resuscitation is secondary to a cardiac event, birth asphyxia is the most important cause leading to neonatal resuscitation [1]. Globally, birth asphyxia accounts for approximately one-fourth of 4 million deaths each year [2,3]. The proportion of neonatal mortality as a percentage of childhood mortality has steadily increased over the last two decades. In 2017, 47% of mortality under the age of five years worldwide was among newborn infants, compared to 40% in 1990 [4]. During the fetal transition to a neonate, asphyxia can lead to primary apnea, which usually responds to stimulation/airway patency, and if not addressed, could lead to secondary apnea that often requires effective ventilation and advanced resuscitation [5,6,7,8]. If resuscitative efforts are delayed, it could lead to irreversible hypoxic-ischemic insult and, ultimately, cardiac arrest [5,6,7,8]. Thus, establishing effective ventilation remains the single most important step in neonatal resuscitation [1]. The response to effective ventilation is assessed by an increase in heart rate (HR) [1]. The International Liaison Committee on Resuscitation (ILCOR) and neonatal resuscitation program (NRP) recommends initiation of external CC if the HR remains less than 60 bpm after 30 s of effective ventilation [9,10]. The use of electrocardiograms (ECG) to monitor HR since the year 2015 has improved our ability to detect bradycardia in the delivery room [10,11]. Early cord clamping and hypothermia can interfere with physiological transition and increase the risk of persistent bradycardia. The majority of neonatal deaths due to birth asphyxia occur in low- and middle-income countries (LMICs). Resuscitation protocols, such as Helping Babies Breathe (HBB), that cater to the LMICs are effective and are based on ventilatory support only, without recommendations to initiate CC [12]. In contrast to neonatal resuscitation guidelines, pediatric and adult resuscitation recommends CC only in the presence of pulselessness or signs of poor perfusion such as altered mental status or hypotension/shock, although the etiology of bradycardia and arrest is more likely to be cardiac dysfunction in these age groups. This manuscript reviews the available literature, strategies to prevent the progression of bradycardia, and evidence evaluating the role of CC for persistent neonatal bradycardia in the delivery room.

## 2. Discussion

### 2.1. Current Practices of Neonatal and Infant Resuscitation

In the delivery room, the majority of neonates transition successfully and establish lungs as the site of gas exchange without any assistance [1]. The infants who require resuscitation are at higher risk of neurological morbidity and mortality. Global initiatives and improvements in resuscitative strategies are part of the solution to reduce perinatal mortality [13,14,15]. In persistently bradycardic (HR < 60 bpm) newborns, despite effective ventilation, ILCOR recommends CC in the ratio of 3 compressions to 1 breath, with a total of 120 events in a minute, until the HR improves [10].

The HBB program was launched in June 2010 as part of a global alliance and has evolved over the years and shown promising results in LMICs, in the face of millennium development goals to reduce under-five child mortality [14]. The simple algorithm of HBB emphasizes providing ventilation and assessing breathing and HR during resuscitation. If the HR remains slow, HBB recommends continuing ventilation and deciding on advanced care, and does not provide guidelines for CC [12]. Both of these contrasting resuscitation practices for neonatal bradycardia do not have any clinical or translational evidence. In the postnatal age group, Pediatric Advanced Life Support (PALS) recommends performing CC and ventilations in the ratio of 15:2 or 30:2 for bradycardia with signs of poor perfusion [16]. While there are studies evaluating the outcomes in bradycardic pediatric patients after resuscitation, there are no clinical studies in neonates to assess the outcomes after performing CC for bradycardia in the delivery room.

### 2.2. Effective Strategies to Prevent Worsening of Neonatal Bradycardia at Birth

**Figure 1:**
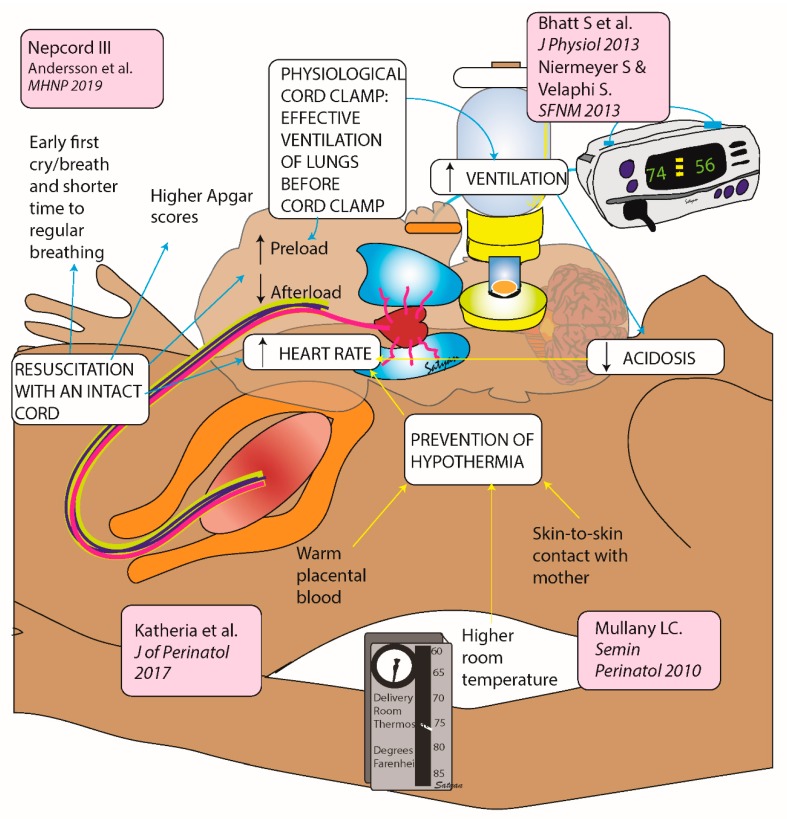
Effective strategies that could prevent neonatal bradycardia. Copyright Satyan Lakshminrusimha.

Recent studies and evaluations of evidence by experts suggest that neonatal bradycardia could be avoided by physiological cord clamping (Figure 1). Delaying the clamping of the cord until respiration/ventilation is established improves the pulmonary blood flow and left ventricular preload, improving the HR and the cardiac output, thus preventing bradycardia [17,18,19,20,21,22,23]. Hypothermia in newborns can increase the incidence of bradycardia and mortality. In newborn infants undergoing whole-body hypothermia for hypoxic ischemic encephalopathy, bradycardia is one of the side effects for which atropine is available on the bedside as a therapeutic option. Physiological cord clamping with skin-to-skin care while an infant is breathing will help to improve cardiovascular stability and hypothermia and could reduce the need for resuscitation in low-resource settings. The current recommendations place emphasis on effective ventilation of the lungs and use of corrective measures to improve gas exchange as the first step in neonatal resuscitation. However, if bradycardia is worsening, initiation of CC distracts the resuscitation team from potentially useful strategies to improve the HR. Thus, severing the umbilical cord in order to provide advanced resuscitation could worsen the transition of a neonate (Figure 1).

### 2.3. Outcomes of Performing CC for Bradycardia in Pediatric in-Hospital Patients

Two large studies have evaluated the outcomes of performing CC and ventilation in pediatric patients with bradycardia. Donoghue et al. included pediatric hospitalized patients from the National Registry of Cardiopulmonary resuscitation from between 2000 and 2008 who received at least 2 min of CC [24]. The study population was categorized as bradycardia with poor perfusion or asystole/pulseless electrical activity (PEA). They found that when CC were initiated for bradycardia with poor perfusion, survival to discharge was higher compared to those patients who received CC for asystole/PEA. Among 738 newborns with bradycardia/poor perfusion in this study, 262 (36%) survived to hospital discharge. In contrast, only 77 of the 301 newborns with asystole/PEA survived to discharge (26%, *p* = 0.002). A recent study by Khera et al., which included post-neonatal pediatric patients from 2000–2016, reported similar results [25]. In their study, children with bradycardia without pulselessness at the initiation of cardiopulmonary resuscitation (CPR) had 70% survival. Children with initial pulselessness had a 37.5% survival. Among those with bradycardia (*n* = 2799), 31% became pulseless after initiation of CPR. Interestingly, among children with bradycardia who received CC with subsequent pulselessness, a longer duration of CC prior to pulselessness was associated with lower survival [25]. Survival was 47% if children progressed to pulselessness following <2 min of CC, 25.2% with pulselessness following 2–5 min of CC, and only 17.7% with pulselessness following >5 min of CC [25]. The authors speculate that CPR measures applied for bradycardia, especially in patients who transition to pulselessness following prolonged CC, may cause ischemic injury, leading to lower survival. These results could be secondary to the nature of patients’ underlying morbidity and the timing and quality of resuscitation. We also speculate the asynchrony of CC in a heart with perfusing rhythm, i.e., bradycardia, could play a role in aggravating ischemia, leading to pulselessness and cardiac arrest during resuscitation.

### 2.4. Research on Performing Chest Compressions during Neonatal Resuscitation

After evaluating evidence in 2010 and 2015, the ILCOR recommends a 3:1 ratio of CC to positive pressure ventilation (PPV), compared to 15:2 or 30:2 in PALS for persistent bradycardia despite effective ventilation in neonates [9,16,26]. The CC are performed effectively by the two-thumb technique to compress the lower third of the sternum to a third of the depth of the anteroposterior diameter of the chest [27]. Use of 100% oxygen is the current recommendation for ventilation during CC until the end of resuscitation or return of spontaneous circulation (ROSC), although the efficacy of this practice still needs evidence [28,29,30]. Research in CC during neonatal resuscitation has so far focused on different techniques of compression, depth of CC, use of sustained inflations, asynchronous ventilation, oxygen concentrations, and use of assisted devices to predict effective CC and ROSC [1,27,31,32,33,34]. The current recommendation to initiate CC for HR < 60/min during resuscitation is probably based on consensus rather than clear evidence. To our knowledge, there are no published studies randomizing neonates or neonatal animal models with bradycardia secondary to perinatal asphyxia to CC vs. no-CC with a primary outcome of ROSC.

### 2.5. Characteristics of Coronary and Cerebral Blood Flow during Asphyxia

Compromised blood flow to the fetus secondary to uteroplacental insufficiency or impaired gas exchange at the lungs postnatally could lead to impaired pulmonary vascular transition, hypoxemia, metabolic acidosis, low systemic perfusion pressures, poor myocardial perfusion, myocardial depression, and ultimately, cardiac arrest [35,36,37].

Dawes et al. described the concepts of “primary apnea” and “secondary apnea” using transitioning rhesus models of asphyxia [5,6]. During primary apnea due to asphyxia, the blood flow is preferentially channeled to the brain, myocardium, and adrenals, which leads to peripheral vasoconstriction, and thus blood pressure is maintained. However, there is a bradycardic response and gasping respirations during this period [38,39,40,41]. With appropriate stimulation with patent airways, a neonate may promptly recover from primary apnea [42]. If the asphyxia insult continues, the gasping respirations cease with a worsening of metabolic acidosis and systemic hypotension, which may eventually lead to cardiac arrest. The response to extensive resuscitation, which includes PPV, CC, and medications following secondary apnea, may depend on the extent of asphyxial insult [42].

The blood flow distribution during asphyxia has been studied extensively in translational models [38,39,40,41]. In fetal lambs who were hypoxic and acidemic, blood flow to the brain, heart, and adrenals increased 2–3-fold [38]. Similar responses were seen when asphyxia was induced by uterine and umbilical blood flow disruption to the fetus [39,41]. During asphyxia, cerebral perfusion happens during the peak antegrade systolic flow, which is followed by a reversal of blood flow during the peak retrograde diastolic flow. Similarly, cardiac perfusion happens during the peak antegrade diastolic flow, followed by a loss of perfusion during the peak retrograde systolic flow. We have studied the effect of asphyxia and HRs on cerebral and coronary perfusion in a perinatal ovine asphyxiated model (HR > 100 bpm, 100–80 bpm, 79–60 bpm and <60 bpm, CC during cardiac arrest, during and post-ROSC, Table 1) [43]. A modest reduction in HR did not result in reduced carotid or coronary flow [43]. During asphyxia-induced neonatal bradycardia, unless the HR is extremely low or zero, compensatory cardiac mechanisms appear to maintain cerebral perfusion and coronary pressure. Under these circumstances, it remains unclear if performing asynchronous CC will increase blood flow to the heart and brain.

### 2.6. Positive Pressure Ventilation and Its Effect on Cerebral and Coronary Perfusion during Neonatal Bradycardia

**Figure 2:**
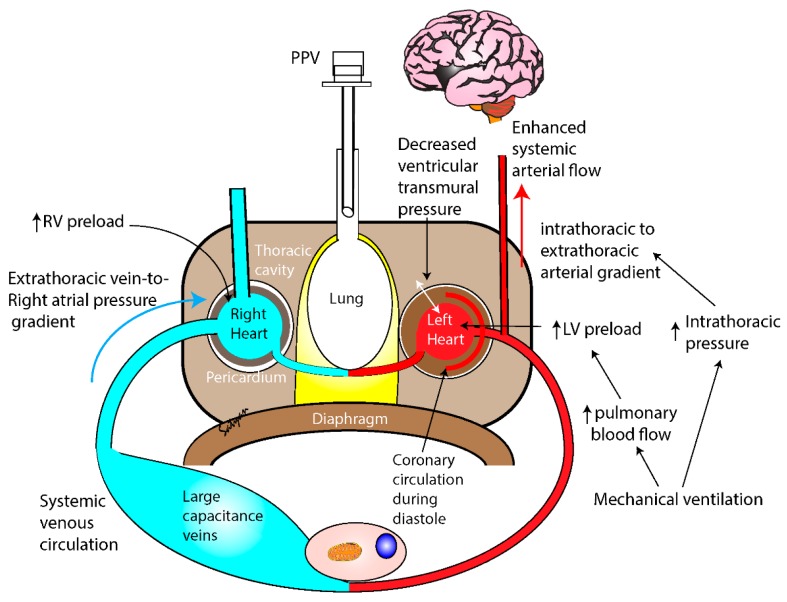
Effect of positive pressure ventilation (PPV) on pulmonary and systemic hemodynamics during neonatal bradycardia. Aeration of lung increases pulmonary blood flow facilitating gas exchange, maintaining left ventricular preload, and enhancing cerebral blood flow during systole and coronary blood flow during diastole. The pressure gradients caused during PPV enhance venous return, maintaining right ventricular preload and pulmonary circulation. (LV-left ventricle, RV- right ventricle). Copyright Satyan Lakshminrusimha.

The most effective step during neonatal resuscitation is ventilation of the lungs [1]. Aeration of the lungs leads to a decrease in pulmonary vascular resistance (PVR) and an increase in pulmonary blood flow (PBF), facilitating gas exchange [44]. The improvement in PBF is of prime importance to maintain the left ventricular output especially in asphyxia that worsens the PVR further. Use of supplemental oxygen during resuscitation may further improve PBF, which could potentially improve systemic oxygenation [28,45,46]. Previous translational studies have shown that PPV not only improves gas exchange but also naturally enhances cerebral blood flow due to higher intrathoracic pressure. The transthoracic pressure gradient during ventilation may improve ventricular preload, leading to improved coronary perfusion especially during neonatal bradycardia [47,48]. Increased intrathoracic pressure with PPV decreases ventricular transmural pressure and increases the intrathoracic-to-extrathoracic pressure gradient and improves cerebral blood flow (Figure 2). Thus, rhythmic PPV can enhance right ventricular and left ventricular preload and enhance pulmonary and systemic circulation in the presence of bradycardia.

### 2.7. Effect of Chest Compressions during Bradycardia

**Figure 3:**
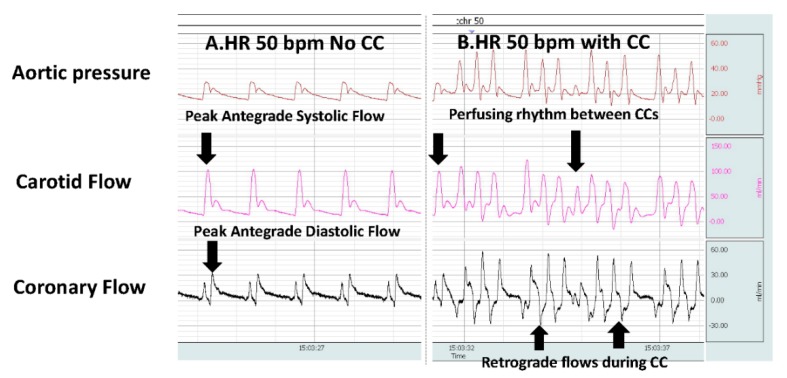
Hemodynamic changes during asphyxia-induced bradycardia and subsequently during chest compressions. The BIOPAC snapshot shows the aortic pressure and carotid and coronary flows. (**A**) During asphyxia-induced bradycardia (HR 50 bpm) with peak systolic carotid and peak diastolic coronary flows. (**B**) When CC is performed during bradycardia, note the retrograde flows. Also note the inherent perfusing rhythm between CCs. Copyright Praveen Chandrasekharan

With the use of an electrocardiogram in the delivery room, the detection rate of bradycardia (HR < 60 bpm) increases and the likelihood of performing CC will be higher during neonatal resuscitation [11]. In an attempt to revive the failing heart and to maintain blood flow to the brain, external CC is provided if the HR remains <60 bpm [1]. Optimal external CC along with ventilation is expected to deliver at least 30% of baseline perfusion preferentially towards the brain and the heart in adult models [27,35,37]. The blood flow to the brain during asphyxia occurs during the peak systolic phase and to the heart during the peak diastolic phase (Figure 3). During CC in a cardiac arrest model in lambs with a patent ductus arteriosus, we have observed retrograde cerebral blood flows during the diastolic phase of CC [49]. Sobotka et al. evaluated a near-term ovine model where asphyxia was induced by delaying the initiation of ventilation until the mean arterial pressure dropped to 20 mmHg. In this study, initiation of CC led to retrograde flows with lower mean carotid blood flows prior to epinephrine administration [36]. In our pilot study with coronary flows in an asphyxiated bradycardia model with an open ductus, when CC were initiated for a HR <60 bpm, we observed retrograde coronary flows as shown in Figure 3 [43].

### 2.8. Could Performing Chest Compressions in the Presence of Perfusing Rhythm Lead to Cardiac Injury and Arrest?

**Figure 4:**
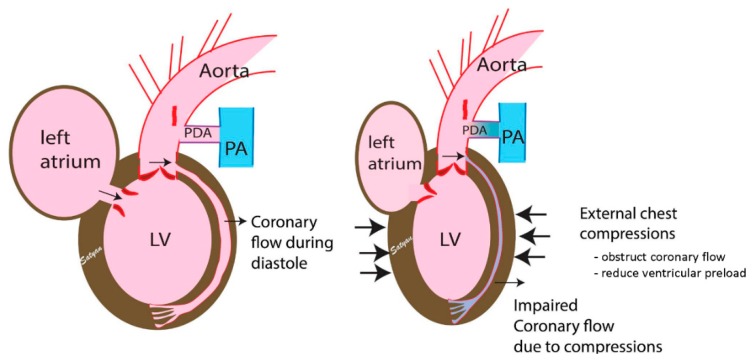
The illustration shows the effect of external cardiac compression during the “diastolic phase” of neonatal bradycardia. The coronary flow and ventricular filling occurs during diastole. Performing asynchronous external chest compressions could interfere with coronary perfusion and impair ventricular filling. (PA—pulmonary artery, PDA—patent ductus arteriosus). Copyright Satyan Lakshminrusimha.

**Figure 5:**
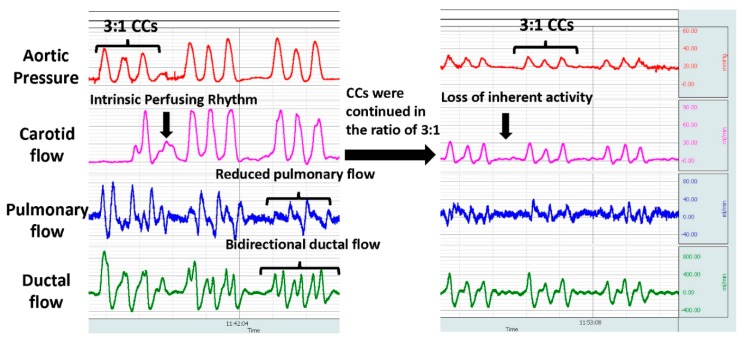
The BIOPAC snapshot shows the aortic pressure and carotid, pulmonary, and ductal flows during chest compressions (CC). Note the initial inherent heart activity in between CCs is lost while CC is continued with cardiac arrest. In addition, the pulmonary flow is reduced along with ductal shunting. Copyright Praveen Chandrasekharan.

With an inherent perfusing rhythm, initiating CC could create atrioventricular dyssynchrony and may worsen perfusion to the myocardium, leading to cardiac arrest (Figure 4). The mechanism of blood flow during external chest compressions during neonatal resuscitation is based on the “cardiac pump” and “thoracic pump” theories [50]. The “cardiac pump” theory postulates that blood is pumped secondary to the squeezing action of the heart, while the “thoracic pump” theory postulates that blood flows from the thorax because intrathoracic pressure exceeds extrathoracic vascular pressure, which serves as a driving force for antegrade blood flow. These theories are based on CC provided during complete cardiac arrest in an adult model without a ductal shunt. The validity of these theories while providing external CC during neonatal bradycardia is not known. Ventricular filling and myocardial perfusion occur during the peak phase of diastole, and cerebral perfusion occurs during the peak phase of systole. During mild-to-moderate asphyxia, blood flow to the brain and heart are preserved during bradycardia (Table 1). External CCthat are asynchronous to innate cardiac activity during bradycardia could potentially interfere with the natural systolic and diastolic phases, which could cut short the ventricular filling, affecting the preload (Figure 4). With impaired ventricular filling, the peak systolic cerebral flows may be reduced. This is further complicated by the presence of ductus arteriosus, resulting in lower diastolic pressures and decreased coronary flow. Previous studies have found that end-diastolic volume is an important determinant of fetal cardiac output [51,52,53]. The Frank–Starling relationship is functional in a fetal heart, and preload is reduced when compressing the heart in diastole [51,52,53]. Chest compressions also have the potential to reduce pulmonary blood flow due to increased intrapulmonary pressure. Furthermore, our observations could also explain the findings of Khera et al. in the pediatric age group, where prolonged CC for bradycardia prior to pulselessness was associated with reduced survival (Figure 5). Duration of CPR correlates with elevation of Troponin I, a marker of cardiac injury, providing an association between CC and cardiac ischemia [54].

### 2.9. Is an HR of <60 bpm an Ideal Cut-Off to Initiate CC in Neonates?

In neonates, HR is a major determinant of cardiac output, given the significantly higher HR range compared to children and adults [55,56]. While the goal of CC is to revive the failing heart and maintain cerebral perfusion to avoid hypoxic ischemic injury, the ideal HR cut-off to initiate CC remains unknown. In the presence of cardiac arrest or pulselessness without perfusion, CC can be beneficial by providing some antegrade coronary, cerebral, and pulmonary flow. However, during bradycardia, asynchronous CC can potentially be harmful. More evidence is required to establish an optimal cut-off for initiating CC that would ideally improve both cerebral and coronary perfusion, leading to faster and increased rates of ROSC in neonates.

## 3. Conclusions

With the ILCOR recommendations to use an electrocardiogram in the delivery room, the incidence of bradycardia and the risk of performing CC has increased threefold [11]. Resuscitation with an intact cord, physiological cord clamping with the establishment of ventilation before cord clamping, and prevention of hypothermia are strategies that could prevent progression from bradycardia to arrest, especially in a low-resource setting. In contrast to adult and pediatric CPR, where CC is indicated only for pulselessness or poor perfusion, neonatal resuscitation recommends CC for bradycardia irrespective of perfusion status. However, uncertainty remains regarding the following issues: (1) the effect of asynchronous CC performed during resuscitation when there is an inherent HR (HR < 60 bmp); (2) the benefit/harm of performing CC during bradycardia on blood flow to the heart, lung, and brain; (3) the optimal HR cut-off to initiate CC. Further research is necessary to compare PPV alone vs. PPV + CC for the management of neonatal bradycardia with perfusion

## Figures and Tables

**Table 1:** Peak blood flows during asphyxia [43].

Parameters	Heart Rate (bpm)	Peak Diastolic Coronary Flow (mL/kg/min)	Peak Systolic Carotid Flow (mL/kg/min)	Peak Systolic Pulmonary Flow (mL/kg/min)
Baseline	167 ± 21	17 ± 12	52 ± 27	110 ± 40
HR > 100	140 ± 28	20 ± 13	46 ± 17	117 ± 41
HR 100–80	89 ± 6	30 ± 16 *	28 ± 13	86 ± 67
HR 79–60	72 ± 6	20 ± 10	58 ± 30 *	86 ± 68
HR < 60	51 ± 7	22 ± 11	26 ± 11	86 ± 52
CC	100 ± 15	5 ± 2	20 ± 13	19 ± 15

Data presented as mean ± SD. * *p* < 0.001 by ANOVA. CC—chest compressions. Heart rate (HR) ranges are during asphyxia.
